# Supplementary material for: PLVAP as an Early Marker of Glomerular Endothelial Damage in Mice with Diabetic Kidney Disease
Source: Int J Mol Sci. 2023 Jan 6;24(2):1094. doi: 10.3390/ijms24021094 (PMC9865597; doi:10.3390/ijms24021094)
Supplement: Supplementary file 1 [file ijms-24-01094-s001.zip › ijms-2008194-supplementary.pdf]

Table S1. Antibodies for immunohistochemistry.

Primary Antibody	Dilution	Supplier/ID	Secondary Antibody	Dilution	Supplier/ID
rat anti-PLVAP/ MECA-32	1:50	Santa Cruz/ sc-19603<br><br>King A et al. Tumor-homing peptides as tools for targeted delivery of payloads to the placenta. *Science advances* 2: e1600349 (2016).<br><br>Langlet F et al. Tanycytic VEGF-A boosts blood-hypothalamus barrier plasticity and access of metabolic signals to the arcuate nucleus in response to fasting. *Cell Metab* 17: 607-17 (2013).	donkey anti-rat IgG, Cy™3	1:500	Jackson ImmunoResearch/ 712-165-150
rabbit anti-ERG	1:100	Abcam/ ab92513<br><br>Andrade J et al. Control of endothelial quiescence by FOXO-regulated metabolites. *Nat Cell Biol* 23: 413-423 (2021).<br><br>Nitzsche A *et al.* Paladin is a phosphoinositide phosphatase	donkey anti-rabbit IgG, Alexa Fluor® 555	1:500	Invitrogen/ A31572

		regulating endosomal VEGFR2 signalling and angiogenesis. *EMBO Rep* 22: e50218 (2021).			
rabbit anti-CD31	1:100	Abcam/ ab182981<br><br>Grosse L *et al.* Defined p16High Senescent Cell Types Are Indispensable for Mouse Healthspan. *Cell Metab* 32: 87-99.e6 (2020).<br><br>Le TM *et al.* Hydrostatic pressure can induce apoptosis of the skin. *Sci Rep* 10: 17594 (2020).	goat anti-rabbit IgG, Alexa Fluor® 647	1:500	Life Technologies/ A21244
guinea pig anti-nephrin	1:100	Progen/ GP-N2<br><br>Wanner N et al. DNA Methyltransferase 1 Controls Nephron Progenitor Cell Renewal and Differentiation. *J Am Soc Nephrol* 30: 63-78 (2019)	donkey anti-guinea pig IgG, Alexa Fluor® 647	1:500	Jackson ImmunoResearch/ 706-605-148
mouse anti-α8Integrin	1:100	Santa Cruz/ sc-365798	goat anti-mouse IgG,	1:500	Thermo Fisher/ A21147

		Majumdar U et al. Single-cell RNA-sequencing analysis of aortic valve interstitial cells demonstrates the regulation of integrin signaling by nitric oxide. *Front Cardiovasc Med* 9: 742850 (2022).<br><br>Xie Y et al. Quantitative in situ proximity ligation assays examining protein interactions and phosphorylation during smooth muscle contractions. *Anal Biochem* 577: 1-13 (2019).	Alexa Fluor® 555		
goat anti-ICAM2	1:100	Invitrogen/ PA5-47939<br><br>Generated using a mouse specific antigen (Mouse myeloma cell line NS0-derived recombinant mouse ICAM-2 Ser20-Gln222)	donkey anti-goat IgG, Alexa Fluor® 647	1:500	Life Technologies GmbH/ A-21447

Table S2. Parameters for automatic histological analysis.

**Staining**	**Preprocessing**	**Thresholding**	**Postprocessing**	**Coverage**
PLVAP	Gaussian blur (2), histogram adaption, median filter (radius=1)	Fixed threshold, 800 in GIPR^{dn} mice and STZ mice, adaptive threshold, 1.2 x (median + SD of intensity) in BTBR *ob/ob* mice	Exclude dark outliers	90%
CD31	Background subtraction (radius=20)	Fixed threshold, 1,000	-	100%
α8Integrin	Background subtraction (radius=20)	Fixed threshold, 500	-	80%
ERG	Histogram adaption, background subtraction (radius=20)	Fixed threshold 2,000	-	95%
nephrin	Histogram adaption, background subtraction (radius=10)	Fixed threshold, 2,000	-	50%
ICAM2	median filter (radius=2)	Fixed threshold, 2,500	-	90%

Table S3. Dataset of glomerular PLVAP expression [% of glomerular area].

number of values	**control mice**	**GIPR^{dn} mice**	**control mice**	**BTBR *ob/ob* mice**
1	0.97	3.68	2.58	4.05
2	1.30	1.77	3.22	6.43
3	0.72	2.57	2.53	3.40
4	0.45	2.12	1.43	4.54
5	0.46	2.33	1.64	3.97
6			2.16	4.21
mean	0.78	2.49	2.26	4.43
standard deviation (SD)	0.36	0.72	0.66	1.05
standard error of the mean (SEM)	0.16	0.32	0.27	0.43

Table S4. Dataset of glomerular CD31 expression [% of glomerular area].

number of values	**control mice**	**GIPR^{dn} mice**	**control mice**	**BTBR *ob/ob* mice**
1	38.25	40.08	48.49	56.27
2	32.52	43.61	37.97	45.14
3	51.50	53.21	53.74	50.69
4	54.84	49.75	63.23	44.91
5	39.23	38.19	29.26	49.17
6			50.11	40.93
mean	43.27	44.97	47.13	47.85
standard deviation (SD)	9.47	6.37	11.97	5.38
standard error of the mean (SEM)	4.24	2.85	4.89	2.20

Table S5. Dataset of glomerular ERG-positive cells [% of glomeruli].

number of values	**control mice**	**GIPR^{dn} mice**	**control mice**	**BTBR *ob/ob* mice**
1	36.62	41.37	19.17	23.60
2	32.90	36.16	15.81	34.31
3	34.05	35.82	27.91	25.91
4	39.04	28.17	26.86	22.28
5	35.98		24.61	23.48
6			22.98	
mean	35.72	35.38	22.89	25.91
standard deviation (SD)	2.38	5.44	4.65	4.87
standard error of the mean (SEM)	1.06	2.72	1.90	2.18

Table S6. Dataset of glomerular α8Integrin expression [% of glomerular area].

number of values	**control mice**	**GIPR^{dn} mice**	**control mice**	**BTBR *ob/ob* mice**
1	53.39	52.01	47.15	55.23
2	47.75	53.06	38.23	51.12
3	53.55	53.01	56.82	56.31
4	59.02	52.73	63.41	53.76
5	49.57	47.09	42.75	54.27
6			54.34	49.47
mean	52.66	51.58	50.45	53.36
standard deviation (SD)	4.34	2.54	9.41	2.58
standard error of the mean (SEM)	1.94	1.14	3.84	1.05

Table S7. Dataset of glomerular nephrin expression [% of glomerular area].

number of values	**control mice**	**GIPR^{dn} mice**	**control mice**	**BTBR *ob/ob* mice**
1	55.07	56.14	39.00	43.43
2	53.57	50.50	43.65	42.63
3	56.85	53.04	48.97	45.59
4	57.55	54.62	49.82	39.74
5	52.07	54.31	46.83	38.64
6			49.54	38.46

mean	55.02	53.72	46.30	41.42
standard deviation (SD)	2.27	2.11	4.26	2.91
standard error of the mean (SEM)	1.01	0.95	1.74	1.19

Table S8. Dataset of glomerular area [μm^2].

number of values	**control mice**	**GIPR^{dn} mice**	**control mice**	**BTBR *ob/ob* mice**
1	2873.74	4064.28	2293.59	3509.04
2	3051.24	4902.28	2425.52	5374.62
3	3395.30	4036.50	2063.74	4174.39
4	2882.89	4359.74	1967.64	4800.98
5	3059.64	3998.23	2473.83	4042.15
6	3475.85	4046.22	2544.06	4926.52
7	3351.46	4309.25		
8	2873.25			
9	2993.95			
10	3064.17			

mean	3102.15	4245.22	2294.73	4471.28
standard deviation (SD)	225.57	322.97	233.10	682.67
standard error of the mean (SEM)	71.33	122.07	95.16	278.70

Table S9. Dataset of glomerular cell density [cells per μm^2 glomerular area].

number of values	**control mice**	**GIPR^{dn} mice**	**control mice**	**BTBR *ob/ob* mice**
1	0.01282	0.01372	0.01186	0.00844
2	0.01371	0.01239	0.01204	0.00970
3	0.01508	0.01291	0.01270	0.00947
4	0.01527	0.01115	0.01114	0.00924
5	0.01257	0.01013	0.01139	0.00924
6			0.01097	0.00834

mean	0.01389	0.01206	0.01168	0.00907
standard deviation (SD)	0.00125	0.00143	0.00064	0.00056
standard error of the mean (SEM)	0.00056	0.00064	0.00026	0.00023

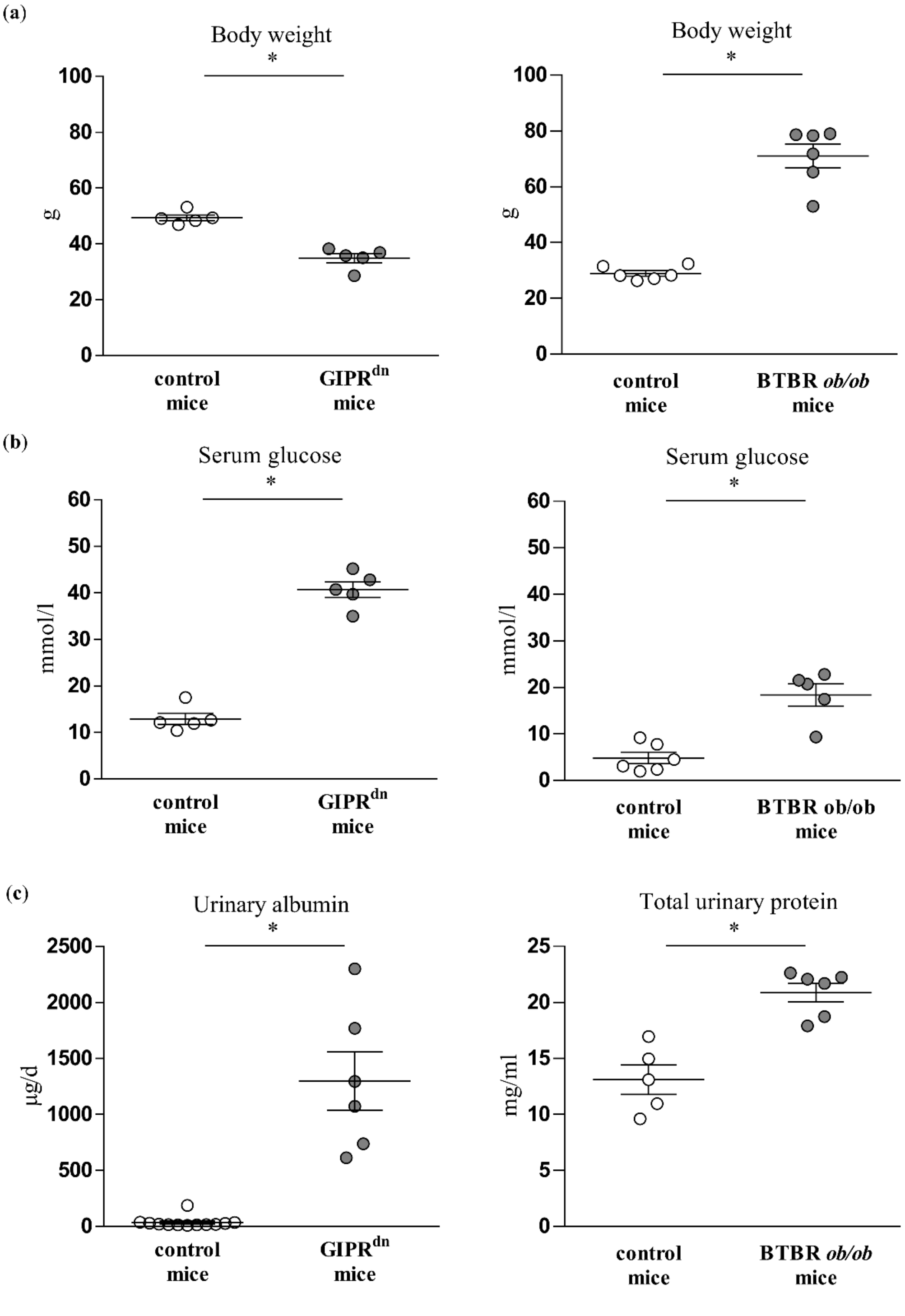

Figure S1. Body weight (**a**), serum glucose (**b**), and proteinuria (**c**) in mouse diabetic kidney disease models compared to their corresponding healthy control mice shortly before the time points of histological analysis. For GIPR^{dn} mice, body weight was determined and blood samples were collected on the day of sacrifice. The mice were placed in metabolic cages three days before sacrifice to collect urine. Blood and urine samples from BTBR *ob/ob* mice were collected on the day of sacrifice. Scatter dot plot with means and SEM, * $p < 0.05$, $n = 5$-12.

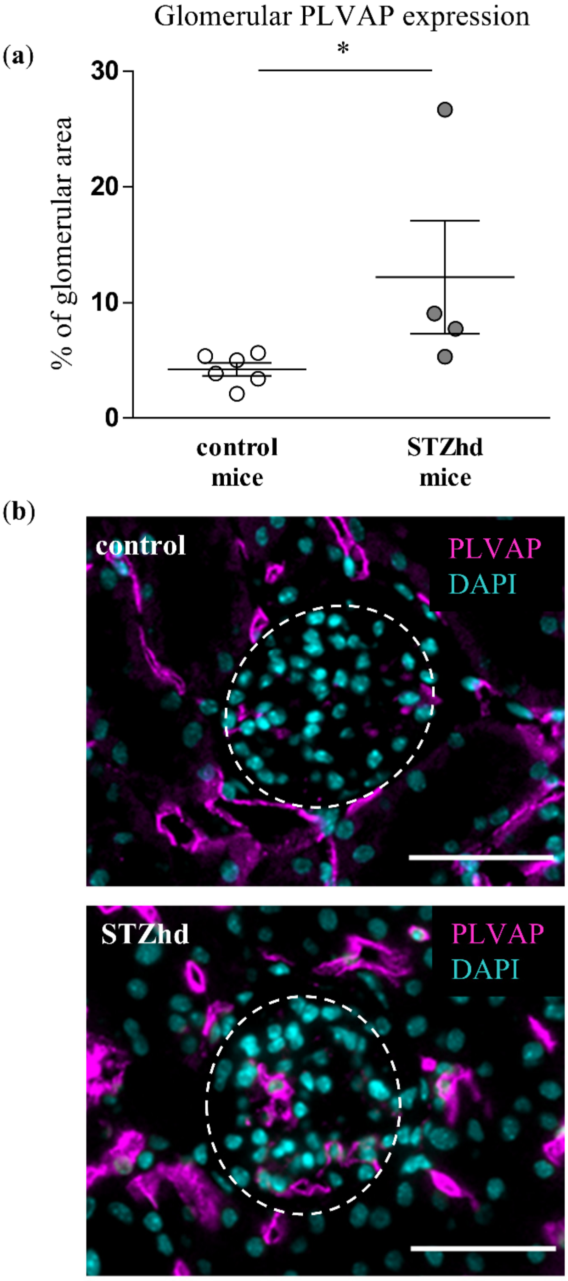

Figure S2. Analysis of plasmalemma vesicle-associated protein (PLVAP) in streptozotocin-treated diabetic mice. (**a**) Automatic histological evaluation of PLVAP-positive area in glomeruli of high-dose streptozotocin (STZhd)-treated mice (Diabetes mellitus type 1 model) compared to their corresponding healthy control mice. Scatter dot plot with means and SEM, * $p < 0.05$, $n = 4–6$. (**b**) Representative images of histological kidney sections stained for PLVAP (magenta) and the nuclear marker 4′,6-diamidino-2-phenylindole (DAPI, cyan). Dashed line borders the glomeruli. Scale bar: 50 µm.

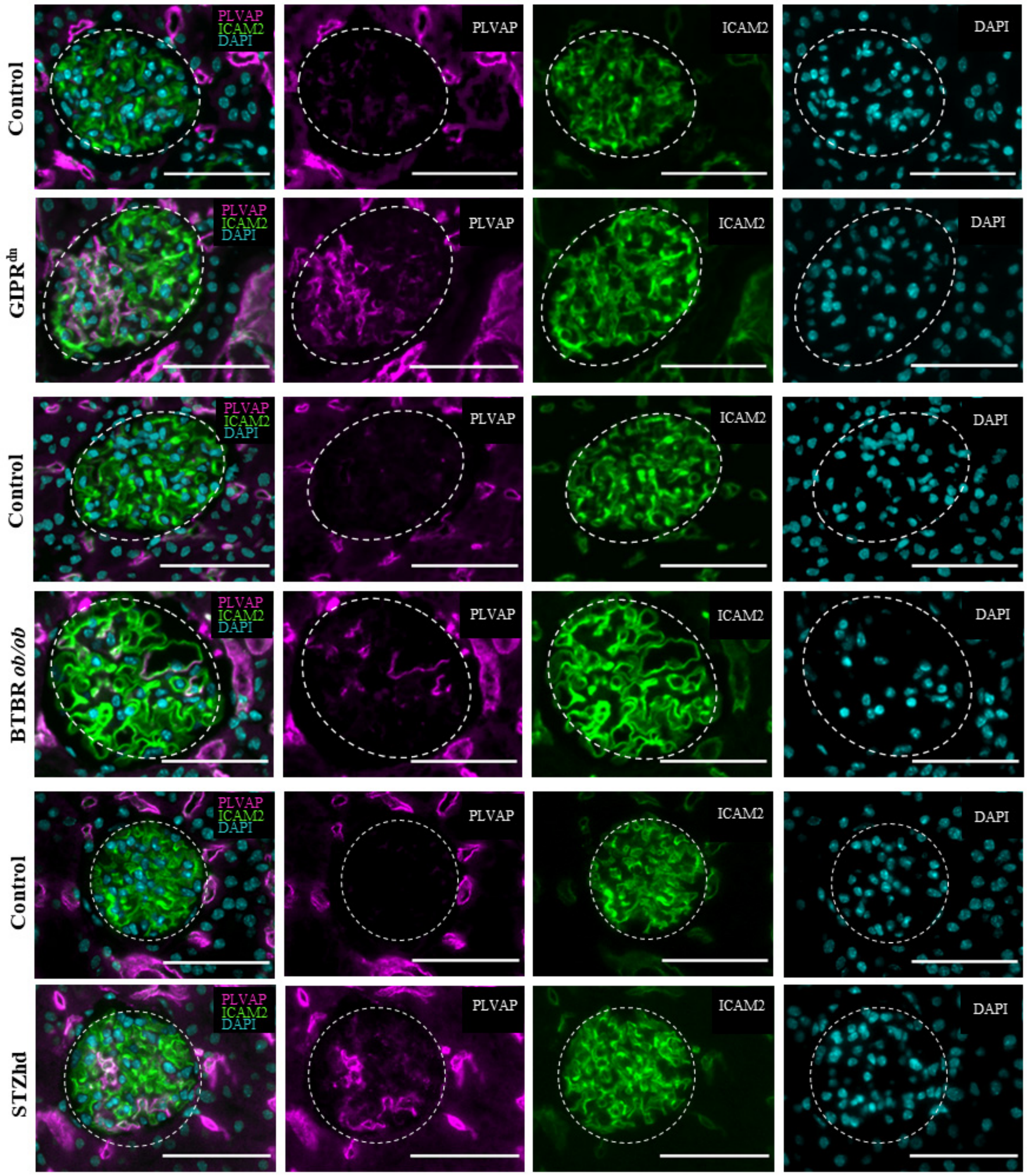

Figure S3. Analysis of PLVAP and intercellular adhesion molecule 2 (ICAM2) glomerular expression in diabetic kidney disease models. Representative images of histological kidney sections from GIPR^{dn} mice, BTBR *ob/ob* mice, high-dose streptozotocin (STZhd) treated-mice, and their corresponding healthy control mice stained for PLVAP (magenta), ICAM2 (green), and the nuclear marker 4´,6-diamidino-2-phenylindole (DAPI, cyan). Dashed line borders the glomeruli. Scale bar: 50 µm.

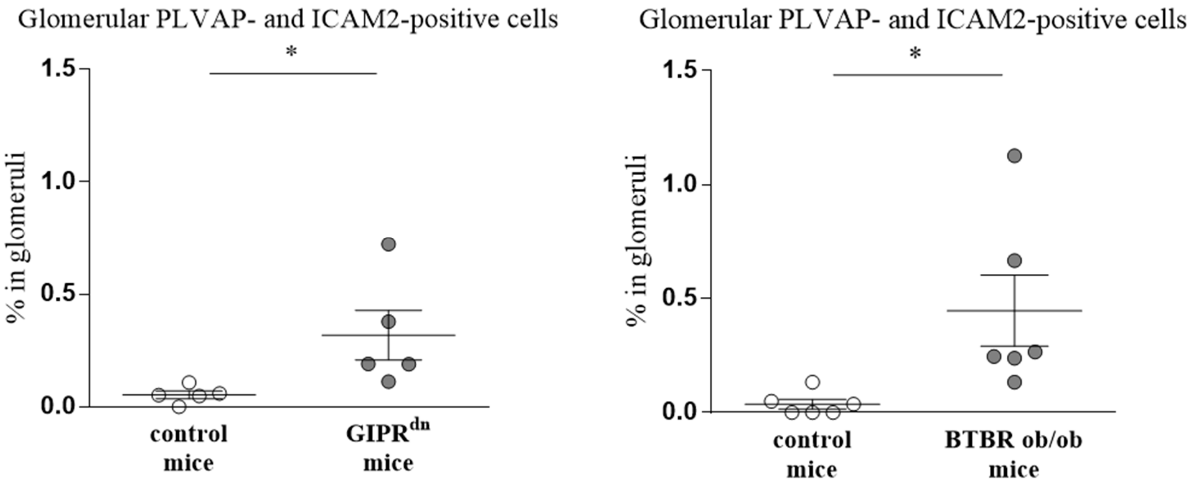

Figure S4. Analysis of co-localisation of PLVAP and ICAM2 in diabetic kidney disease models. Automatic histological evaluation of PLVAP- and ICAM2-positive cells (+/+) in glomeruli of GIPR^{dn} mice and BTBR *ob/ob* mice compared to their corresponding healthy control mice. Scatter dot plot with means and SEM, * $p < 0.05$, $n = 5–6$.

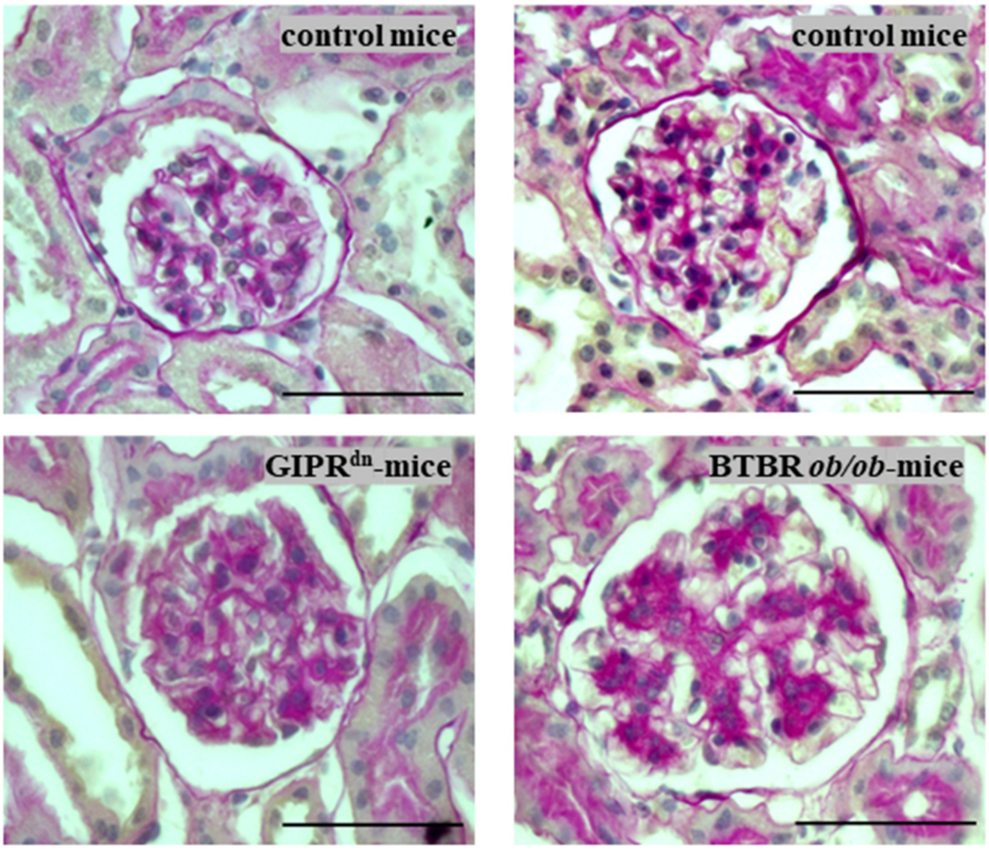

Figure S5. Representative images of periodic acid-Schiff-stained (PAS) glomeruli in diabetic kidney disease models. PAS histological kidney sections from $GIPR^{dn}$ mice (left, Diabetes mellitus type 1 model) and BTBR *ob/ob* mice (right, Diabetes mellitus type 2 model) and their corresponding healthy control mice. Scale bar: 100 µm.

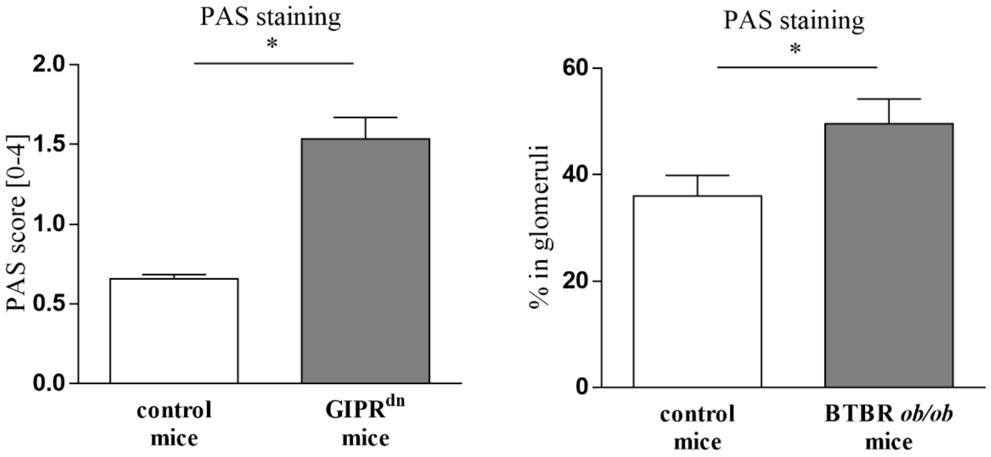

Figure S6. Analysis of periodic acid-Schiff-stained (PAS) glomeruli in diabetic kidney disease models. Histological evaluation of PAS-positive area in glomeruli in GIPRdn mice (left) and BTBR *ob/ob* mice (right) compared to their corresponding healthy control mice. Means and SEM are shown, * $p < 0.05$, $n = 6–11$.
